# Nutritional Approach to Diabetic Sarcopenia: A Comprehensive Review

**DOI:** 10.1007/s13668-025-00637-0

**Published:** 2025-03-19

**Authors:** Gül Eda Kılınç, Yeliz Vergi

**Affiliations:** 1 Faculty of Health Sciences, Department of Nutrition and Dietetics, Ondokuz Mayıs University, Samsun, Turkey; 2 Faculty of Health Sciences, Department of Nutrition and Dietetics, Mersin University, Mersin, Turkey

**Keywords:** Diabetes mellitus, Elderly, Nutrition, Sarcopenia

## Abstract

**Purpose of the Review:**

The aim of this review is to discuss and evaluate diabetic sarcopenia (DS) and its relationship with nutrition by discussing the mechanisms of diabetic sarcopenia in detail and comprehensively reviewing the literature.

**Recent Findings:**

Type 2 diabetes (T2DM) affects approximately 25% of people aged 50 years and over and indicates a significant the cost of health for the elderly. Nutrition is an important part of these treatment approaches, and in this review, the literature was comprehensively reviewed, focusing on understanding the mechanisms of DS and discussing its relationship with nutrition. A comprehensive search was conducted on Web of Science, Google Scholar, Scopus, Science Direct, and PubMed from inception up to July 2024. The aim of nutritional treatment for DS is to improve muscle mass, muscle strength and physical performance while improving diabetes-related metabolic risk and glucose levels. In this context, it is important to determine energy intake in individuals with DS according to calorie intake exceeding 30 kcal/kg. For these individuals, a protein intake of at least 1–1.2 g/kg/day is recommended, with an emphasis on the number and timing of meals and a nutritional pattern rich in branched chain amino acids (BCAA). In addition, it is important to adopt a diet rich in antioxidants and to choose diet patterns that contain sufficient levels of macro and micronutrients.

**Summary:**

The Mediterranean diet model can be a good diet option for individuals with DS. Comprehensive studies in this field are needed so that clinicians can make specific dietary recommendations for DS.

## Introduction

Sarcopenia is a geriatric condition characterized by a progressive loss of muscle mass and function and is associated with a variety of adverse health outcomes, including fractures, functional decline, and death [1]. While sarcopenia commonly affects older individuals, it can also be prevalent among certain populations, such as those with cancer, metabolic disorders, kidney dysfunction, and liver disorders. Sarcopenia can also be considered an important prognostic indicator in terms of survival and clinical complications in these patients [2]. Diabetic sarcopenia (DS) is characterized by the loss of muscle mass in individuals with the disease, as opposed to normal muscle mass both histologically and physiologically [3].

Diabetes is a major factor in skeletal muscle loss and affects body composition, specifically visceral fat and decreased muscle and bone mass, in individuals with Type 2 diabetes (T2DM) [4]. In addition, an increase in oxidative stress, a rise in the incidence of malnutrition, and various energy imbalances can be seen due to the inflammatory state that diabetes causes [5]. Sarcopenia and T2DM are correlated in both directions and they increase each other’s risks in a vicious circle. Recently, it has been stated that the mortality rate in T2DM and sarcopenia patients is higher than in other patients. To effectively manage this clinical complication, it is crucial to look at the pathophysiology of both diabetes and concomitant sarcopenia in the elderly, as relatively little attention has been paid to this population [6]. Poor glycemic control, especially in individuals with diabetes, is directly related to muscle loss, decreased strength and overall physical performance. This connection is basically explained by four possible mechanisms. First, insulin is known to play a key role in muscle function by increasing glucose uptake and directly interfering with intracellular glucose metabolism. In this way, insulin resistance may have a negative effect on muscle strength [7]. Second, the anabolic activation pathway known as the Target of Rapamycin (mTOR) metabolic pathway is regulated by insulin resistance. There might be less protein available for protein anabolism as a result of this pathway’s decreased ability to synthesize proteins [8]. Third, muscle protein breakdown, insulin resistance, lipolysis, and nitrogen loss can all be brought on by long-term inflammation-related cytokines associated with T2DM [9]. Finally, ongoing hyperglycemia can cause changes in muscle mass by causing the accumulation of advanced glycation end products (AGES) [10].

In the light of this information, this review focused on understanding the mechanisms of DS and discussing its relationship with nutrition by comprehensively reviewing the literature. This review provides an important contribution to the literature by focusing on the underlying molecular and pathophysiological mechanisms of DS and by addressing nutritional approaches within the framework of these mechanisms. Unlike previous studies in the literature, this article discusses nutritional approaches in DS from a holistic perspective, primarily by detailing the pathophysiological processes. This study comprehensively integrates the relationship between the biological mechanisms involved in the DS process and nutritional interventions, and offers a perspective that highlights the mechanism-nutrition interaction, which has been limited in the previous literature.

## Methods

Despite the lack of systematic approach, a wide search strategy was taken to find suitable information for this review. Using relevant terms unique to each component, the database was searched using four search engines: Web of Science, Google Scholar, Scopus, Science Direct, and PubMed. Only English-language articles were found throughout the search, The inclusion criteria for the search include studies that offer useful information, theoretical frameworks, or empirical data particular to each component of the article. The evaluation process is content-oriented. Studies that lack methodological rigor or are not pertinent to the topic are examples of exclusion criteria.

**Fig. 1:**
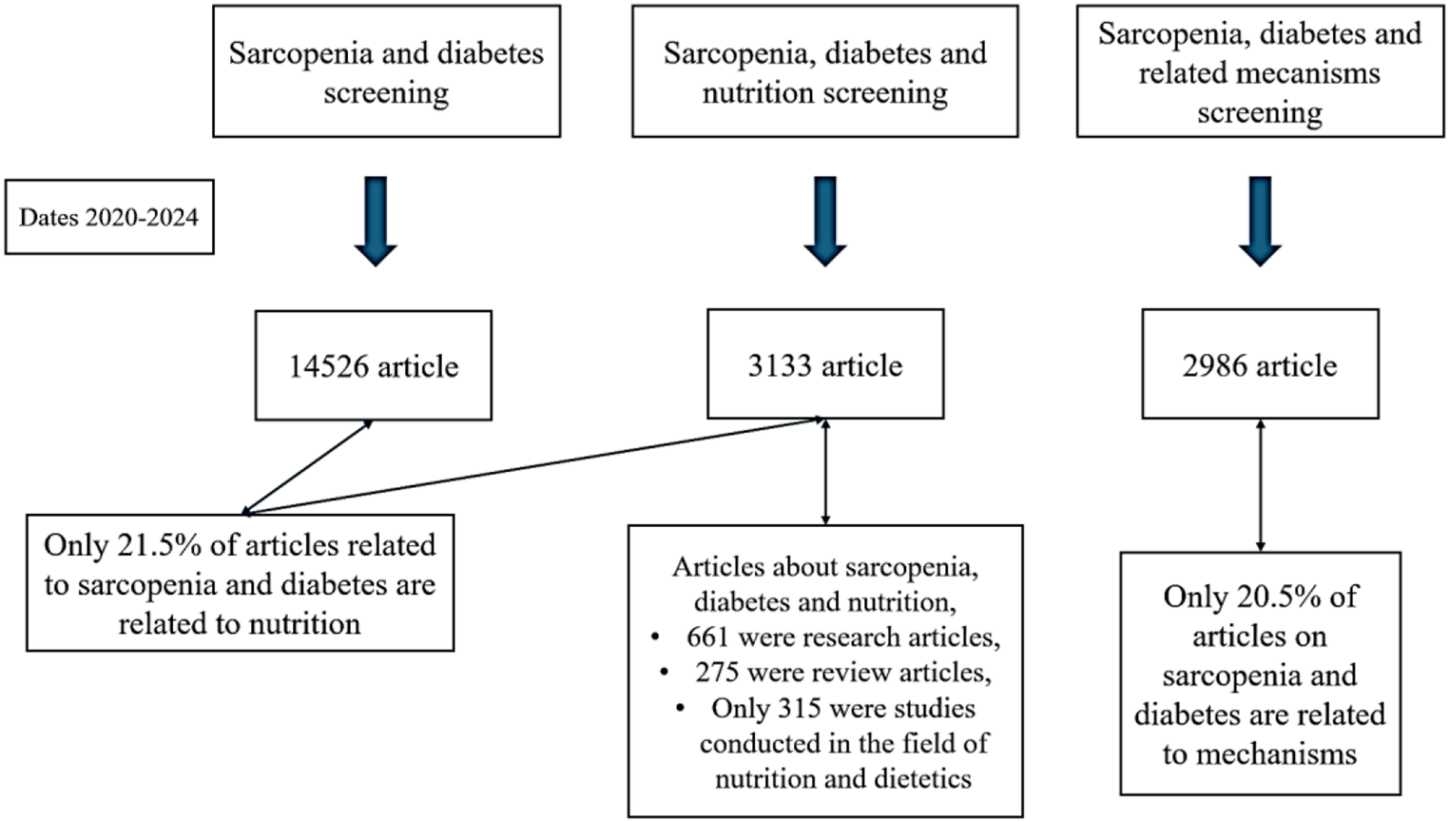
Sub-distribution of studies related to diabetes and sarcopenia. ‘Source of Figure: Authors own creation’

**Fig. 2:**
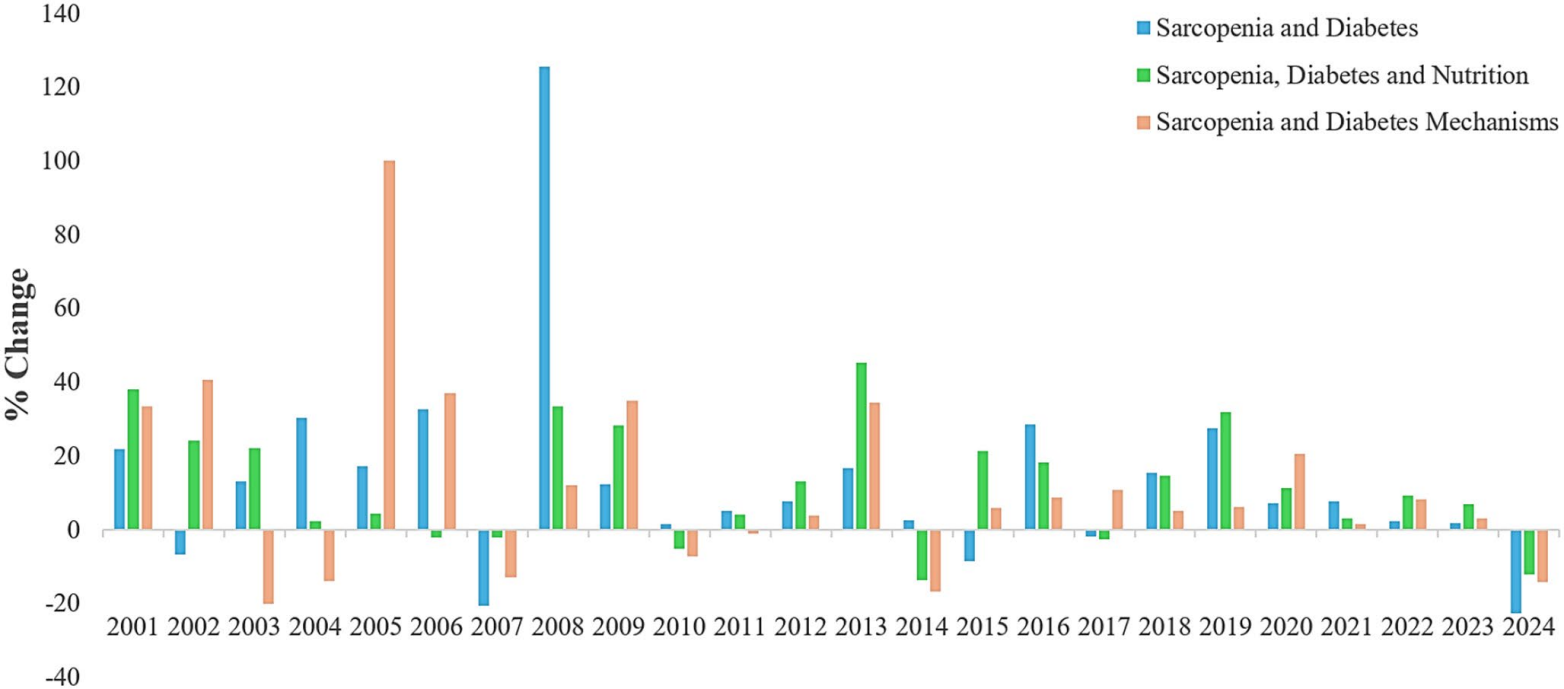
Annual change in general, nutrition-focused and mechanism-based studies on DS between 2000 and 2025. ‘Source of Figure: Authors own creation’. DS: Diabetic Sarcopenia

When the studies related to DS and nutrition were analyzed in Web of Science between 2000–2025 years, searches were first performed with the terms “diabetic sarcopenia”, “diabetes and sarcopenia”, “diabetes and muscle loss” and “nutrition and sarcopenia and diabetes” using the search button. Then, by including the relationship with nutrition, the terms diabetes and sarcopenia and nutrition”, “diabetes and muscle loss and nutrition”, “diabetes and sarcopenia and dietary intervention”, “diabetes and protein intake and sarcopenia” and “diabetes and amino acids and muscle loss” were searched. The search was further expanded with the terms “diabetes and sarcopenia and oxidative stress”, “diabetes and sarcopenia and inflammation”, “diabetes and muscle loss and insulin resistance” and “diabetes and sarcopenia and mitochondrial dysfunction”. Diagrams for searches from the database are shown in Figs. 1 and 2. According to a search of the Web of Science database, nutrition-based studies constitute a relatively small portion of the total volume. This indicates that research focusing on nutrition studies should be increased.

### The Metabolic Pathophysiology for Diabetic Sarcopenia

**Fig. 3:**
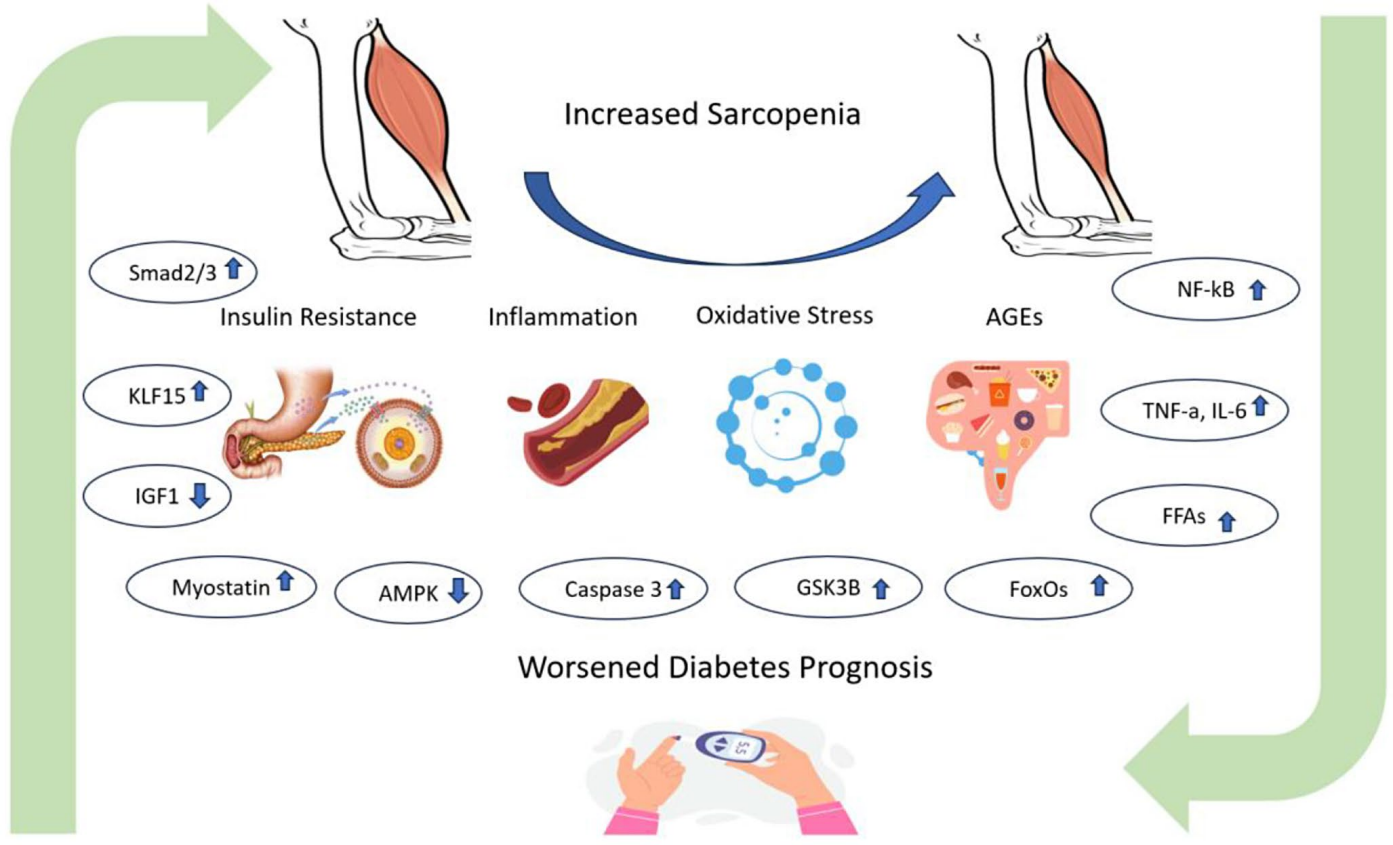
Interaction of sarcopenia and diabetes, ‘Source of Figure: Authors own creation’

Many factors play a role in the concept of DS, and important associated mechanisms are shown in Fig. 3.

#### Insulin Resistance (IR)

The main mechanism of T2DM is insulin resistance (IR), and one of the target organs of insulin is the skeletal muscle system [11]. In the presence of insulin resistance, protein degradation can be promoted and protein synthesis can be prevented by the impaired insulin mechanism. In this way, decreases in both muscle mass and muscle strength may occur. An IR occurring in the skeletal musculature is a very important factor in the exacerbation of sarcopenia The main proteolytic mechanisms in muscle are the calcium-dependent calpain pathway, the ATP-dependent ubiquitin proteasome pathway (UPP), the lysosomal autophagy pathway, and the caspase hydrolysis pathway. These pathways have direct interactions with the IL6/STAT, FoxO1/3 and TNF&IL6/NFκB myostatin/Smad2/3 signaling pathways [3]. Insulin inhibits the catalytic activity of proteasomes in muscles, and elevated blood sugar levels in the context of insulin resistance can lead to muscular atrophy via the ubiquitin-proteasome proteolytic pathway [12]. Furthermore, through WWP1/KLF15, IR can cause hyperglycemia and muscular atrophy [13].

In an investigation on diabetic animal models, it was reported that hyperglycemia upregulates the skeletal muscle-associated protein KLF15, which is linked to muscle atrophy, by downregulating the E3 ubiquitin ligase WWP1. This, in turn, inhibits the ubiquitin-dependent degradation of KLF15. Thus, the skeletal muscle of diabetic mice increased as a result of increased transcription factor KLF15 and altered gene expression linked to muscle atrophy; mice lacking muscle-specific KLF15 were spared from the effects of diabetes on muscle atrophy [14]. When it comes to treating sarcopenia brought on by insulin resistance, this pathway may hold promise. The production of muscle proteins is primarily regulated by two factors: myostatin and insulin-like growth factor 1 (IGF1) [15]. The suppression of the IGF1-PI3K-Akt-mTOR and SC-Gαi2 pathways mediates the inhibition of protein synthesis [3]. These signaling pathways are essential for the growth and control of skeletal muscle. A key player in this cascade, Akt kinase, sometimes referred to as protein kinase B (PKB), regulates protein synthesis through mTOR, glycogen synthase kinase 3 (GSK3), and the FoxO family of transcription factors for protein synthesis [16]. In the presence of IR, sarcopenia occurs due to inhibition of the mTOR pathway [17]. In a clinical study conducted with elderly Japanese individuals, it was stated that T2DM was associated with possible sarcopenia based on low hand grip strength and IR based on the TyG index. It has been stated that when these occur together, there is a particularly strong relationship with T2DM [18]. Additionally, IR enhances lipolysis and suppresses the growth hormone (GH)-insulin-like growth factor (IGF1) axis, which releases free fatty acids (FFA) to promote protein synthesis in skeletal muscle, when skeletal muscle mass decreases [19]. Furthermore, transforming growth factor β (TGF-β) superfamily member myostatin promotes AMPKα2 phosphorylation and decreases mTORC1 signaling. Dysregulation of protein synthesis and an increase in autophagic proteolysis can result from both processes [20]. It has been demonstrated that myostatin-induced apoptosis is caused by the p38-caspase pathway being activated, which increases protein degradation [21]. Skeletal muscle atrophy could result from IR-induced hyperinsulinemia via raising the myostatin content [22].

#### Inflammation

It has long been hypothesized that persistent tissue inflammation may play a role in metabolic illness. In the past 20 years, a lot of significant research that offer essential knowledge for the field of immunometabolism have been published. For instance, in rodents, tumor necrosis factor (TNF-α) induces glucose intolerance [23]. TNFα and other pro-inflammatory cytokines can inhibit the effects of insulin by activating key intracellular signaling molecules like JNK and IKKb that are part of the inflammatory signaling system [24]. Nuclear factor kB (NF-kB) is nuclear translocated as a result of IKKb activation, which also increases the expression of inflammatory mediators such as chemokines and cytokines [25]. When diabetes and inflammation coexist, PI3K-Akt mediates the downregulation of protein anabolism while stimulating ubiquitin to upregulate protein catabolism [26]. Furthermore, it is asserted that increased pro-inflammatory levels substances, such as TNF-α, CRP, and IL-6 are harmful to the growth and functionality of muscles [27]. Additionally, compared to non-diabetic controls, older people with T2DM saw a greater loss of leg muscle and strength over a three-year period [28].

#### Oxidative Stress

It is believed that oxidative stress, which arises when there are more reactive oxygen species (ROS) and less antioxidants present, plays a major role in aging and the development of chronic diseases [29]. The presence of oxidative stress is the critical factor in the process of muscle atrophy with inflammation, mitochondrial dysfunction, decreased protein synthesis and increased proteolysis. In particular, oxidative stress is the main factor that triggers skeletal muscle atrophy. In the early stages of muscle atrophy, oxidative stress is activated and can be regulated by various components. Although the mechanisms of oxidative stress in the development of muscle atrophy are not fully elucidated, some mechanisms are emphasized [30]. Even while T2DM is linked to higher levels of oxidative stress, hyperglycemia in particular can cause higher levels of ROS to be produced [31]. Sarcopenia may result from the activation of the ubiquitin-proteasome system, which speeds up the breakdown of muscle proteins. In diabetic rats, it has been demonstrated that elevated oxidative stress hinders DNA structure and activity, muscle repair, and cell differentiation [32]. Mechanistically, high ROS generation in myoblasts can suppress MyoD expression and increase NF-κB, which would prevent myogenic differentiation. Thus, there may be a greater chance of protein breakdown [33]. Moreover, non-lysosomal Ca 2 + regulated calpains may be triggered by loss of Ca 2 + homeostasis in diabetics as a result of elevated oxidative stress (an additional indicator of T2DM), and aberrantly activated calpain may cause muscle atrophy by activating the ubiquitin-proteasome pathway [34].

### Advanced Glycation End-products (AGEs)

The generation of ROS can cause the upregulation of intracellular AGEs, among many other effects in diabetes. AGEs normally build up gradually in the human body over the course of a lifetime, mostly in tissues with sluggish metabolisms. Diabetes mellitus, renal failure, and other chronic illness conditions speed up this process. Inflammatory cells, endothelial cells, and vascular smooth muscle cells all have receptors for advanced glycation end products (RAGEs). When AGEs bind to RAGEs, proinflammatory cytokines and growth factors are released from macrophages, ROS generation occurs from the endothelium, and the endothelium and macrophages have a procoagulant effect [35]. In T2DM patients, hyperglycemia is linked to aging, IR, and persistent AGE accumulation. It has also been demonstrated that AGE accumulation causes skeletal muscle atrophy and dysfunction in both animal models and human research [36].

### The Nutritional Therapeutic Strategy for Sarcopenia In T2DM

#### Energy

A generally high prevalence of sarcopenia is observed in individuals with T2DM [37, 38]. Worsening of nutritional status may cause this prevalence to increase. Calorie restriction is known to be an effective strategy for weight loss, especially in overweight and obese individuals. However, while weight loss may lead to remission of T2DM in individuals with diabetes, it may lead to significant losses in muscle mass. At this point, while providing weight loss through calorie restriction in individuals with T2DM, insufficient protein intake should also be prevented [39].

**Fig. 4:**
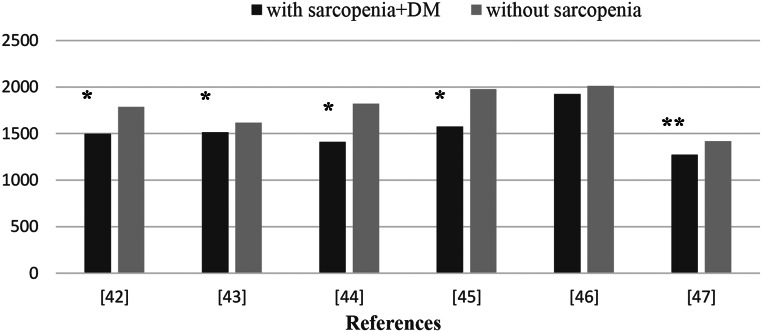
Evaluation of energy intake in individuals with T2DM with and without sarcopenia in epidemiological studies. * *p* < 0.05; ** *p* < 0.001. Source of Figure: Authors own creation’. T2DM: Type 2 diabetes

Although there is no specific recommendation for DS, daily energy intake is recommended as 25–30 kcal/kg/d in Japan for patients with T2DM [40]. According to the ESPEN geriatric guide, it is stated that daily energy intake in the elderly should be above 30 kcal/kg in order to reduce malnutrition and the resulting sarcopenia [41]. Although there are different results in the literature, it has been repeatedly reported that energy intake is significantly lower in individuals with T2DM and sarcopenia. Therefore, adequate energy intake is recommended for elderly diabetic patients other than morbidly obese patients. There are very few studies on the energy intake of individuals with sarcopenia and diabetes, and existing epidemiological studies are summarized in Fig. 4 [42–47].

#### Protein

Ensuring adequate protein intake is very important in the development and maintenance of muscle mass. According to the ESPEN guideline, at least 1–1.2 g is recommended for elderly individuals, and higher intakes can be increased in the presence of comorbidities [41]. In addition, protein recommendations for T2DM are compatible with those recommended for the general population and are 1.0–1.2 g/kg/day, while patients with diabetic nephropathy are recommended to limit their protein intake to 0.8 g/kg/day. It is also stated that corrected body weight should be used for obese and overweight individuals [48].

In addition to protein intake amounts, dietary protein sources are also very important. In this context, eggs appear to be a very good source of protein as an example. Eggs, both whole and white, are useful as dietary treatments because of their favorable effects on muscle hypertrophy and preservation of function, as demonstrated by scientific research [49]. It was found that there were improvements in glucose intolerance and grip strength after 8 weeks in sarcopenic obesity model mice supplemented with dried egg in the diet [50]. In studies evaluating the effects of whey protein supplements on muscle strength, it has been stated that whey protein supplements have positive effects on muscle strength in sarcopenic or weak and healthy older adults. Additionally, it has been stated that muscle strength increases with whey protein intake higher than 20 g [51, 52]. In addition, comprehensive meta-analysis studies have shown that vitamin D (VD) supplementation with whey protein has positive effects on muscle strength and physical function, as well as improvements in glucose balance [53, 54].

BCAA supplementation on T2DM are unknown, despite the fact that BCAAs are known to impact insulin signaling, kynurenine metabolism, and myofibrillar protein synthesis. In a study, which looked at the impact of giving senior people with T2DM either 7.5 g of soy protein or 8 g of BCAA supplementation for 24 weeks, it was discovered that the soy protein group’s knee extension muscle strength grew significantly while the BCAA group’s group showed a tendency to increase. Results showed that BCAA supplementation improved glycemic control while not affecting skeletal muscle mass [55]. Another similar study found that short-term administration of BCAAs was associated with short-term positive effects on sarcopenic parameters [56]. In addition, protein quality and timing of protein intake are also important issues in DS.

**Fig. 5:**
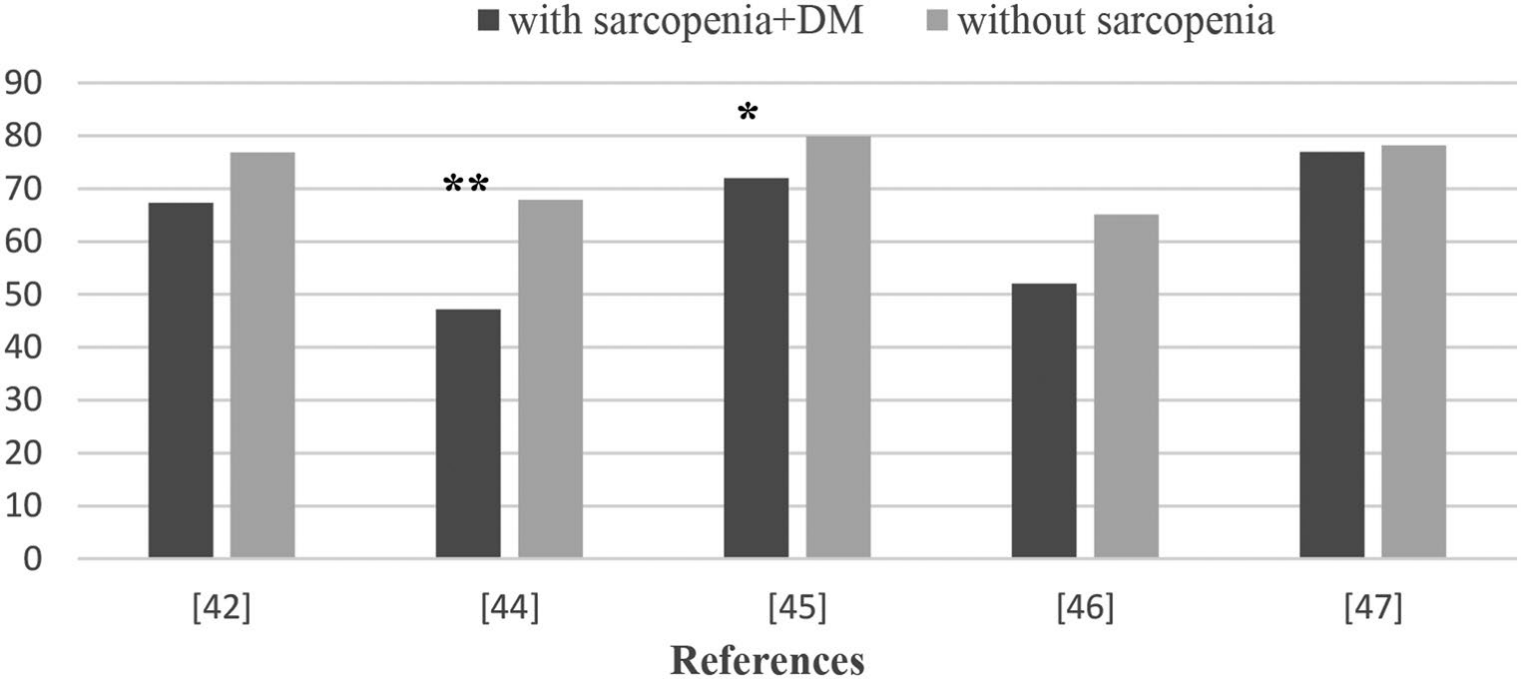
Evaluation of protein intake in individuals with T2DM with and without sarcopenia in epidemiological studies. * *p* < 0.05; **p < 0.001. Source of Figure: Authors own creation’. T2DM: Type 2 diabetes

While it is stated that animal proteins are more effective than plant proteins in muscle protein synthesis, it is also important to know that animal-based proteins may be associated with kidney dysfunction, one of the advanced complications of diabetes [57]. As long as adequate protein consumption is ensured, muscle maintenance and repair may not rely heavily on plant or animal-based protein [58]. In addition, the number and timing of meals may also be important in DS. For example, it is stated that consuming more protein at breakfast may prevent sarcopenia than consuming more protein at dinner [59]. However, comprehensive studies are needed to clearly explain the relationship between protein quality or timing of protein intake and sarcopenia in elderly patients with diabetes. Protein intake appears to be generally low in individuals with DS. Therefore, adequate protein intake is recommended for elderly diabetic patients to maintain and maintain muscle strength. Epidemiological studies on protein intake in individuals with T2DM and sarcopenia are summarized in Fig. 5 [42, 44–47].

#### Polyunsaturated Fatty Acids

The prevalence of T2DM is increasing markedly in almost all countries, and all major guidelines emphasize the importance of dietary therapy. However, the relationship between PUFA and the incidence of T2DM is unclear. In a meta-analysis study, total PUFA intake was associated with an increased incidence of T2DM in Europe and Australia, but with a decreasing incidence in Asia. It has been stated that there is no linear relationship between PUFA intake and the incidence of T2DM [60]. Despite this, it is stated that the diabetic picture progresses towards diabetic retinopathy, especially with the changes that occur with the intake of five n-6 PUFAs (LA, GLA, EDA, DGLA and AA) [61]. It is stated that n-3 PUFAs have beneficial clinical effects on glycemic control, triglyceride levels and inflammation in patients with T2DM, and n-3 PUFA intake is supported in the management and prevention of T2DM complications [62].

Regarding sarcopenia, it has been stated that n-3 PUFA intake is low in older adults with sarcopenia, and in general, while n-3 PUFA intake has a positive effect on sarcopenia parameters, a negative relationship between n-6 PUFA intake and sarcopenia has been determined. However, it is noted that the specific properties of different types of PUFAs (e.g., short-chain vs. long-chain) and the duration of intake and their effects on health may also play an important role [63]. Similarly, in case of sarcopenia, it has been stated that n-3 PUFA supplementation has a positive effect on general body muscle mass and muscle strength [64]. When saturated fatty acids were replaced isocalorically with PUFA, it was observed that the sarcopenia risk score of participants with protein intake < 1.1 g/Body Weight (BW) decreased [65]. On the other hand, it is reported that n-3 PUFA has an anti-inflammatory and anabolic effect on muscle mass and prevents muscle catabolism in DS. Additionally, it has been said that there is little chance of benefit from using n-3 PUFA to reduce inflammation and oxidative stress and enhance systemic insulin resistance in older individuals with IR [66]. Interventions on diabetes, sarcopenia, and PUFA intake are applied alone or in combination with other nutritional factors and exercise, and results can range from insignificant to beneficial. Therefore, it is difficult to develop comprehensive conclusions and recommendations for DS and PUFA intake, considering the heterogeneity in dose amount, duration of interventions, assessment tools and varying intervention methods.

Examining interventional studies, it was shown that 1.2 g/day n-3 PUFA (720 mg EPA and 480 mg DHA) supplementation for six months had a moderate effect on gait speed but no effect on body composition or strength in 126 elderly persons 65 years of age and older [67]. In comparison to a corn oil placebo, older persons who took 4 g/day n-3 PUFA supplements (1.86 g EPA, 1.5 g DHA) experienced a 2.3 kg gain in hand grip strength and a 3.6% increase in thigh muscle volume [68]. In another similar study, compared to olive oil placebo, a 4% increase in muscle mass was detected in elderly individuals who took 5 g/day fish oil, excluding those who consumed fish more than once a week or took n-3 supplements, as a result of BIA measurement after 3 months [69]. In another three-month trial, it was stated that 1.3 g/day n-3 PUFA supplementation (660 mg EPA, 440 mg DHA) had no effect on body composition, hand grip strength and walking speed evaluated by BIA in individuals aged ≥ 65 years [70]. As a result, it is recommended to restrict SFAs (< 7% of total calorie intake) by including PUFA-rich ingredients in the diet in individuals with DS to prevent complications brought by these diseases in addition to diabetes and sarcopenia [71]. More studies and evidence are needed for additional supplement recommendations for PUFAs.

#### Micronutrients

Based on clinical studies, it is stated that VD has an effect on the mechanism of diabetes by playing a regulatory role in insulin secretion, beta cell survival and calcium flux within beta cells [72]. VD is a steroid hormone produced in the body, and the dietary contribution to the daily VD requirement is not more than 20% of the total. It is stated that VD supplementation may improve HbA1c and insulin response in T2DM patients [73]. Proximal muscular weakness and VD deficiency are related, and VD supplementation is much sought after as a preventative and therapeutic measure for sarcopenia. Though the exact processes underlying the association between VD and sarcopenia remain unclear, it is believed to be mediated by the VD receptor through its effects on protein synthesis and gene transcription, in addition to its possible anti-inflammatory function [74]. Most of the research on VD and muscle health is intervention trials, but mostly in combination with protein and exercise training. This shows that it is very difficult to isolate the effect of VD due to the combination of VD supplementation with other interventions [75]. In the VD levels examined in blood samples, 0.11 kg more contraction force was determined with every 10 units of 25(OH) increase, and a moderate relationship was detected between VD and skeletal muscle mass [76]. It is also stated that the survival rate in individuals with sarcopenia with severe VD deficiency is quite low [77]. In addition, the optimal range of VD is still a matter of debate. While levels below 10 ng/mL are universally considered inadequate for VD, the significance of levels between 10 and 30 ng/mL remains unclear [78]. Related studies in patients with DS continue the same inconclusive pattern [79]. As a result, optimization strategies should be discussed and further research is needed to clearly state the effect of VD intake in diabetic elderly patients [80]. Aside from this, it’s critical to incorporate antioxidant-rich foods in the diet, particularly to protect against the oxidative and inflammatory damage that sarcopenia and diabetes cause. More specifically, increased blood concentrations of selenium, carotenoids, and vitamin E were linked to decreased muscle strength, whereas higher intakes of lycopene, lutein + zeaxanthin, and total carotenoids were linked to increased grip strength [81]. Muscle strength and physical performance were positively correlated with vitamin C sufficiency, according to a cross-sectional study on older Japanese women [82]. To comprehend how dietary antioxidants affect the results of muscle health in people with DS, other methods are required. Specifically, taking into account the function of whole foods that are inherently rich in antioxidants and the possibility of whole diet approaches to encourage their intake may prove to be a more fruitful approach. Apart from this, it is very important to get adequate levels of magnesium, calcium and B group vitamins in the diet, which are known to have positive effects on both diabetes and sarcopenia [83, 84].

It has also been stated that there are important connections between polyphenols and diabetes and sarcopenia [85]. In a study examining whether tea polyphenols improve sarcopenia in elderly T2DM model rats, 300 mg/kg tea polyphenols were supplemented and as a result of the study, it was stated that tea polyphenols can improve sarcopenia in elderly T2DM model rats and may be effective on the regulation of mitochondrial quality control through mechanism pathways [86]. In a meta-analysis study that aimed to summarize the evidence on the effect of polyphenol supplementation on muscle mass, muscle strength and physical performance in individuals with sarcopenia, pooled evidence was presented that treatment with polyphenols may have a beneficial effect on muscle mass in sarcopenic subjects [87]. In a systematic review, it was stated that flavonoids may have great potential in treating sarcopenia [88]. Another study has indicated that tea catechins in particular may be beneficial for skeletal muscle performance, muscle atrophy and movement, and may also protect against secondary disorders resulting from sarcopenia, such as cachexia, weakness and sarcopenic obesity [89]. In a study conducted in diabetic mice, a diet containing 0.04% resvaratrol for 8 weeks was found to result in increased muscle function, decreased levels of ubiquitin, muscle MuRF-1 and LC3-II, and cleaved caspase-3, increased mitochondrial biogenesis, decreased mitophagy, and decreased excessive mitochondrial fusion and fission [90]. Resvaratrol supplementation at 3 g/day for 12 weeks was found to increase SIRT1 expression and p-AMPK to AMPK ratio in adults with T2DM [91].

In addition, it is stated that muscle strength is very important for cardiovascular diseases, which are one of the most common secondary diseases caused by diabetes [92, 93] and a study in which oligonol supplementation, a polyphenol formula that helps naturally increase blood flow to support healthy aging, cardiovascular health and weight loss, was given at 200 mg/kg for 10 weeks, indicated that it attenuated ROS-related inflammation and prevented oxidative damage in an in vitro model of T2DM [94].

In addition to all these, it should not be ignored that all chemicals and drugs, including antioxidants, as well as natural products, can cause toxicity when taken in high doses. Antioxidants are found in many foods, especially fruits and vegetables, and can also be synthesized artificially in laboratories. In recent years, emphasis has been placed on effective non-toxic natural compounds with antioxidant activity, and it is mentioned that the use of plant-derived antioxidants may be a suitable alternative in addition to endogenous antioxidant defense systems [95–97]. Studies have shown that antioxidants can realize their potential protective roles by strengthening the enzymatic and non-enzymatic antioxidant systems and can also reduce the toxicity of common drugs with frequent toxic effects [98–101]. Especially when considering the drugs used in the treatment of T2DM and their toxicity status, drugs containing various compounds from different pharmacological classes with different mechanisms of action, side effect profiles and toxicities are considered. These can be classified as oral antidiabetic drugs, hypoglycemic agents (sulfonylureas and benzoic acid derivatives) or antihyperglycemic agents (biguanides, α-glucosidase inhibitors and thiazolidinediones) [102]. Studies also indicate that diabetes mellitus is a risk factor for drug toxicity and especially nephrotoxicity [103–105]. In addition, it is emphasized that anti-diabetic drugs have different effects on skeletal muscle mass, strength and performance and should be examined in terms of sarcopenia risk [106]. The effect of antidiabetic drugs on sarcopenia is also an ongoing research topic, and some drugs in particular may have uncertain effects. It has been stated that long-term use of some antidiabetic drugs, especially sulfonylurea drugs such as glinide, gliclazide and glibedi, may be associated with muscle loss or injury. It has also been stated that these drugs play a role by inhibiting the KATP channel to stimulate insulin secretion, and this mechanism is associated with rapid skeletal muscle atrophy. Conversely, some antidiabetic drugs are also thought to be promising in preserving muscle quality [107]. Although the mechanisms by which insulin increases muscle protein anabolism at the basis of the diabetes mechanism are not yet fully understood, it is stated that this effect is mostly through mRNA translation and may be related to the increase in microvascular participation, blood flow and amino acid delivery to skeletal muscle and the decrease in protein degradation to a large extent [108]. A study of Japanese patients found that insulin therapy may attenuate the decline in muscle strength in the lower extremities but not in the upper extremities. The study suggested that this may support the clinical use of insulin to reduce the risk of sarcopenia in patients with T2DM [109]. Another study found that insulin helped preserve muscle mass but had no effect on muscle function assessed by handgrip strength [110]. In a study examining the effects of metformin on muscle mass, the intervention was performed for 10 weeks and it was stated that there was no significant change [111]. In contrast, a cohort study found that men treated with metformin lost significantly less total or appendicular lean mass compared to those with untreated diabetes or those treated without metformin [112]. It is important to emphasize that patients with diabetes are more likely to develop sarcopenia, but the differences between studies are insufficient to clarify and make a clear statement about the link between antidiabetic drugs and sarcopenia.

#### Mediterranean Diet

Lower rates of death from all causes have been associated with high levels of adherence to the Mediterranean diet in the elderly population. A nutritious diet rich in vegetables, fish, olive oil, and wine is the Mediterranean diet [113]. In a study investigating the relationship between the Mediterranean diet and sarcopenia in patients with T2DM, It was discovered that a high incidence of adherence to the Mediterranean diet was favorably correlated with a decrease in muscle weakness, strength, and physical performance [114]. The Mediterranean diet has been shown to promote or directly play a critical role in the preservation of skeletal muscle due to its rich content of micronutrients with anti-oxidant properties and anti-inflammatory potential [115]. Additionally, a healthy diet with > 30% and at least 15% of total calories coming from fats (> 10% from saturated fatty acids) and protein and increased physical activity are positively associated with muscle strength and physical activity [116]. In conclusion, the Mediterranean diet and healthy eating styles may be beneficial in preventing DS, but further studies are needed.

#### Other Diet Habits

It has been determined that high protein intake increases muscle strength, physical performance and muscle mass in elderly patients with T2DM [117]. It has been determined that high-energy diets similarly have positive effects on sarcopenia and positively affect muscle components [42, 45, 118]. Conversely, it has been reported that recreational exercise combined with a moderately calorie-restricted diet in older individuals with diabetes can reduce the expression of atrophy-associated myokines and increase the expression of the anti-inflammatory gene IL-15 [119]. High dietary antioxidant intake in individuals with T2DM has been associated with lower fasting blood sugar, abdominal obesity, and sarcopenia [120]. It has also been stated that a slow eating rate adversely affects sarcopenia parameters in individuals with T2DM [121, 122]. In addition, there are also studies stating that various diet and healthy lifestyle interventions do not have any effect on the muscle components of individuals with diabetes [123, 124].

Another type of diet is the ketogenic diet, the most basic feature of which is that it contains high fat, adequate protein levels, and low carbohydrates, which are primarily used in the treatment of chronic aging diseases that are difficult to control [125]. It is stated that ketogenic diet treatment is also important for maintaining muscle and muscle development in elderly individuals who may experience sarcopenia and weakness. The ketogenic diet contains approximately 5% carbohydrates and it is stated that long-term ketogenic diet can reduce hyperglycemia and hyperinsulinemia. Further it has been reported that after 12 weeks of combined ketogenic diet and walking exercise interventions in elderly individuals, 5–10% gains in muscle mass and 30–150% improvements in muscle strength were observed [126]. It has been reported that in the presence of sarcopenic obesity, body weight can be effectively reduced by applying a ketogenic diet and lean body mass can be better preserved by combining it with interval training [127]. In a study conducted on mice, it was stated that the ketogenic diet protects oxidative muscle fibers and improves mitochondrial and antioxidant capacity, while allowing mice to better preserve muscle mass and function with age [128]. In a review, it was stated that the long-term effects need to be supported by more research before general recommendations can be made regarding ketogenic and sarcopenia [129]. The benefits of the ketogenic diet for muscles remain controversial and may have different effects on various fiber types, including muscle fiber type ratio. The ketogenic diet can also sometimes cause adverse effects such as cardiac fibrosis [130]. The ketogenic diet regulates glucose sugar and insulin levels and therefore can be claimed to be an effective approach to diabetes [131]. A study conducted with diabetic mice highlighted the protective effects of a 6-week ketogenic diet treatment on muscle mass and strength [132]. Although the ketogenic diet has been accepted as a safe nutritional option in some studies and there is a lot of clinical evidence supporting the use of the ketogenic diet in diabetes, obesity and endocrine disorders, it is emphasized that it should not be forgotten that it can cause serious side effects, including ketoacidosis [133]. Due to the inconsistency of studies related to diabetes and sarcopenia in the literature, further studies are needed to confirm these relationships.

#### Current Guidelines and Future Ventures

**Fig. 6:**
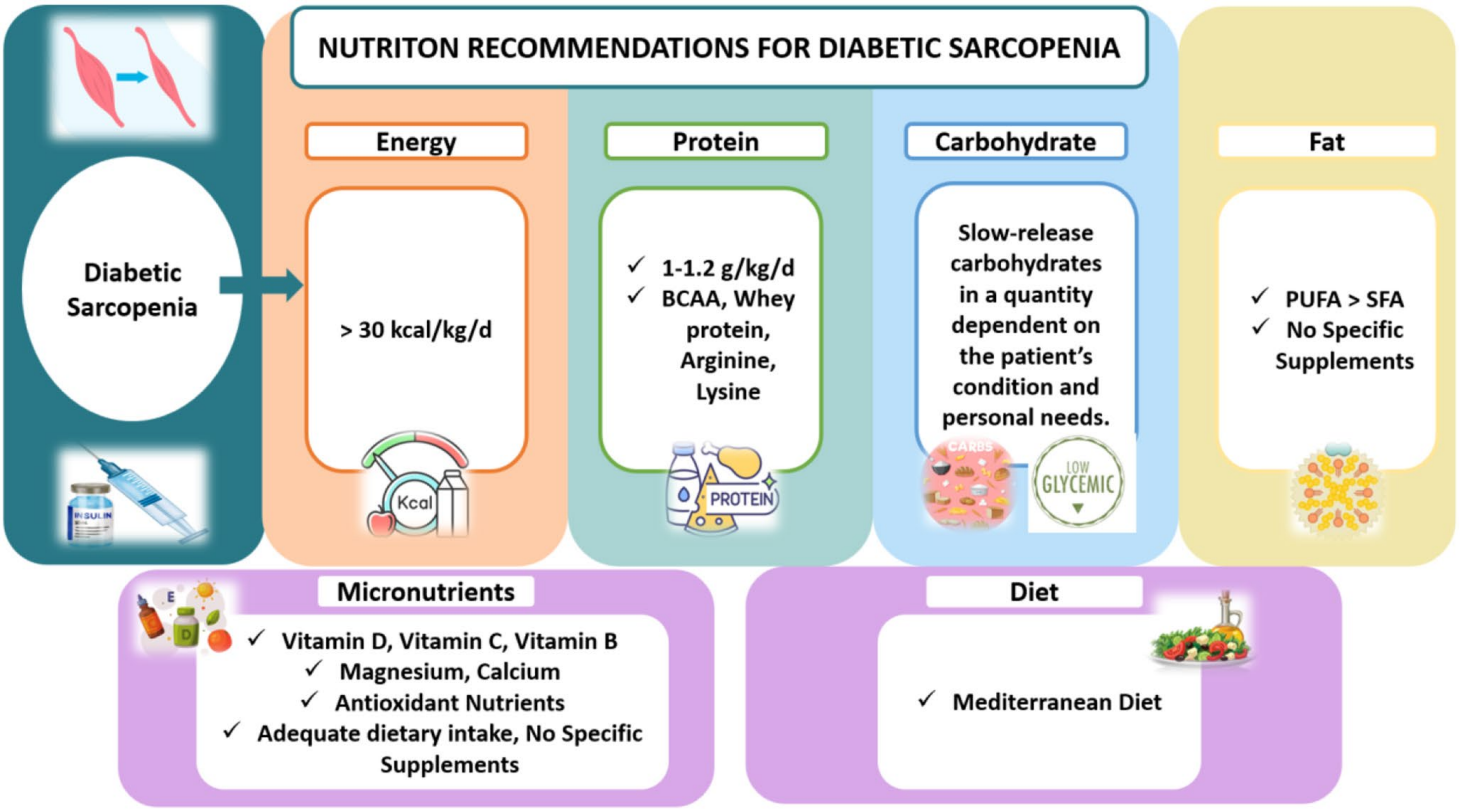
General nutritional recommendations for DS. Source of Figure: Authors own creation’. DS: Diabetic Sarcopenia

As a result of the literature review conducted during this review, although there are many nutrients and diet types that will positively affect muscle strength, physical performance and other related parameters for individuals with DS, there are currently no evidence-based recommendations for the nutritional management of individuals with DS due to the variability of literature results. Therefore, it is important at this stage to be guided by effective and applicable guidelines related to diabetes and sarcopenia. When these guidelines are evaluated in general, it can be stated that recommendations are made by considering diabetes and sarcopenia parameters separately. In this regard, There are suggestions such as consumption of protein supplement/protein-rich diet (> 1 g/kg/day), need to avoid restricted diets, having a daily calorie intake of > 30 kcal/kg, low glycemic targets (HbA1C < 8%), exercise practices, 50–60% of the diet should come from carbohydrates and choices should be made towards low glycemic index and complex carbohydrates in the diet, avoiding unhealthy eating habits, being consistent with meal carbohydrates and timing, administering prandial insulin after meals, tendency towards high energy and protein food preferences, 1000–2000UI/day VD supplementation (25–50 µg/day) for ages 65–75 and 2000–4000 VD supplementation (20–100 µg/day) for > 75 years of age, calcium supplementation of ≥ 1000 IU/day in individuals aged ≥ 65 years to reduce the risk of fractures and falls and malnutrition should not be ignored and malnutrition screening should be performed for individuals with DS in general [40, 41, 134–138]. General nutritional recommendations for DS are outlined in Fig. 6.

## Conclusion

Considering that sarcopenia impairs the quality of life of the elderly, the presence of a serious chronic disease such as diabetes can worsen this situation and lead to an increase in mortality. Diabetes, with its disease mechanism, is not only a chronic disease concept, but also a clinical condition that brings with it many health problems and can irreversibly affect life unless a healthy living conditions are met. Therefore, in the presence of DS, the patient’s quality of life can be seriously affected. In order to alleviate the burden of these severe conditions, it is essential to develop effective management plans, and one of these plans is nutritional therapy. This review focuses on the molecular and pathophysiological mechanisms underlying DS and considers nutritional approaches within the framework of these mechanisms, making an important contribution to the literature. With the nutritional approaches to be applied to individuals with DS, muscle strength, muscle mass and physical performance, which are components of sarcopenia, can be encouraged and maintained, and steps can be taken to improve the quality of life. It should also be kept in mind that diabetes is not a disease that progresses alone, but can progress together with other important diseases that must be approached very seriously, such as cardiovascular diseases, lipid metabolism disorders, high blood pressure or kidney disease, etc. It is thought that it would be useful to examine the relationship between diabetes and sarcopenia together with these diseases, more comprehensively and especially with the support of clinical studies. In addition, when considering the relationship between diabetes and sarcopenia, it should not be forgotten that the condition, age, gender, lifestyle, etc. of diabetic patients can all affect the effect of nutritional therapy. It should be noted that failure to take individual differences into account may lead to limited applicability of the nutritional therapy plan. In this context, it is thought that it would be beneficial to develop new and updated guidelines with ongoing research on DS.

## Key References

**: Outstanding important; *: Important

**R. Kawano, F. Takahashi, Y. Hashimoto, T. Okamura, A. Miki, A. Kaji, R. Sakai, N. Kitagawa, T. Senmaru, S. Majima, H. Okada, “Short energy intake is associated with muscle mass loss in older patients with type 2 diabetes: a prospective study of the KAMOGAWA-DM cohort,” Clinical Nutrition, vol. 40, no. 4, Elsevier, pp 1613–1620, Apr 1, 2021. 10.1016/j.clnu.2021.02.049.
-
  - This is an important prospective cohort study examining the relationship between energy intake and muscle mass loss in elderly patients with type 2 diabetes.
**HA. Giha, OAO. Alamin, MS. Sater, “Diabetic sarcopenia: metabolic and molecular appraisal,” Acta Diabetologica, vol. 59, no. 8, Springer, pp. 989–1000, Aug, 2022. 10.1007/s00592-022-01883-2.
-
  - This article takes a comprehensive approach to diabetic sarcopenia and describes the relationship between diabetes and sarcopenia through the pathways and mechanisms affected during the process.
*M. Kim, T. Kobori, “Association of a Combination of Sarcopenia and Type 2 Diabetes with Blood Parameters, Nutrient Intake, and Physical Activity: A Nationwide Population-Based Study,” Nutrients, vol. 15, no. 23, Mdpı, p. 4955, Nov 29, 2023. 10.3390/nu15234955.
-
  - This article examines the combination of Sarcopenia and Type 2 Diabetes in Japan, where the elderly population is high, by conducting a nationwide population-based study on the subject from blood parameters to physical activity and nutritional intake, from a wide range of perspectives.

## Data Availability

No datasets were generated or analysed during the current study.
